# Serum CD73 activity as a biomarker of hypoxemia in COVID-19 patients

**DOI:** 10.1038/s41598-026-41023-2

**Published:** 2026-02-26

**Authors:** Pierrick Le Borgne, Pascal Bilbault, Raphael Clere-Jehl, Julie Favre, François Lefebvre, Geneviève Ubeaud-Séquier, Fatiha El Ghazouani, Julien Demiselle, Florence Toti, Ferhat Meziani, Gilles Kauffenstein

**Affiliations:** 1 Université de Strasbourg, INSERM, Regenerative NanoMedicine (RNM), UMR 1260, CRBS, 1 rue Eugène Boeckel, Strasbourg, 67000 France; 2 Emergency Department, Hôpitaux Universitaires de Strasbourg, 1 Avenue Molière, Strasbourg cedex, F-67091 France; 3 Biotechnologie et Signalisation Cellulaire, CNRS UMR 7242, Université de Strasbourg, University of Strasbourg, Strasbourg, France; 4 Department of Public Health, University Hospital of Strasbourg, Strasbourg, France; 5 Department of Pharmacy, Strasbourg University Hospital, Strasbourg, France; 6 Service de Médecine Intensive-Réanimation, Nouvel Hôpital Civil, Université de Strasbourg (UNISTRA), 1 place de l’Hôpital, Strasbourg Cedex, 67091 France

**Keywords:** COVID-19, SARS-CoV-2, Ectonucleotidases, CD73, Ecto 5’-nucleotidase, Infection, Innate immunity

## Abstract

Ectonucleotidases regulate extracellular purine signaling and play a key role in inflammation and thrombosis, two major components of COVID-19 pathophysiology. We investigated the enzymatic activities of CD39 (NTPDase1) and CD73 (ecto-5’-nucleotidase) as potential biomarkers of disease severity. Patients with RT-PCR-confirmed COVID-19 were enrolled between August 1st, 2020, and August 1st, 2021, and stratified into mild to moderate (MM, *n* = 55) or severe to critical (SC, *n* = 30) groups according to WHO criteria. Serum samples were collected on days 1, 3, and 7 following Emergency Department (ED) admission. CD39 and CD73 activities were measured by HPLC using etheno-fluorescent nucleotide substrates and compared to healthy volunteers (HV, *n* = 30) matched for age and sex. Serum CD73 activity was significantly elevated in COVID-19 patients compared to HV (MM: 76 ± 9.4; SC: 99 ± 21 vs. HV: 25 ± 2.7 pmol/min/µL; *p* < 0.0001), particularly in patients requiring ≥ 6 L/min oxygen (*p* = 0.005) or with a respiratory rate > 20/min (*p* = 0.02). CD73 activity remained elevated throughout hospitalization. Conversely, febrile patients (≥ 38 °C) and those with CRP ≥ 100 mg/L exhibited significantly lower CD73 activity (*p* = 0.023 and *p* = 0.004, respectively). CD39 activity was undetectable in 28.2% of patients and showed no association with disease severity or outcome. The lowest CD73 levels were observed in patients requiring oxygen therapy for ≥ 30 days and in those who died before day 7. Serum CD73 activity is associated with hypoxemia and oxygen requirements upon ED admission. Conversely, low CD73 activity reflects a hyperinflammatory state and worse outcomes, supporting its potential use in biomarker-based risk stratification for COVID-19.

**Supplementary Information:**

The online version contains supplementary material available at 10.1038/s41598-026-41023-2.

## Introduction

 As of early 2025, the ongoing Coronavirus disease 2019 (COVID-19) pandemic, caused by SARS-CoV-2, has led to over 777 million confirmed cases of COVID-19 and 7 million deaths worldwide^1^. Since the outbreak’s onset, SARS-CoV-2 infection has demonstrated a large spectrum of clinical manifestations^2^. While most patients remain asymptomatic or present with influenza-like symptoms (mild disease), some progress to moderate disease requiring oxygen for viral pneumonia. A minority of patients develop severe or critical disease with complications, such as acute respiratory distress syndrome (ARDS), which may require admission to the intensive care unit (ICU)^3^.

Over the past five years, the world has experienced multiple waves of the COVID-19 pandemic. Despite the widespread availability of vaccines, these successive waves have highlighted the ongoing need for reliable biomarkers to guide clinical decision-making^4^. The search for novel, easily measurable biomarkers with strong diagnostic and/or prognostic value remains crucial in Emergency and Intensive Care Departments particularly in light of evolving viral strains and varying disease severity. While advanced techniques, such as proteomic or metabolomic analyses, provide valuable insights, they are not feasible for routine clinical implementation^5^.

Furthermore, studies on established inflammatory markers - such as interleukin-6, C-reactive protein (CRP), neutrophil-to-lymphocyte ratio, D-dimer, and lymphopenia - have shown inconsistent predictive value^6^. Consequently, a combined approach including clinical symptoms, patient characteristics, imaging, and available biomarkers may offer more accurate predictions of patient outcomes. Despite the emergence of new variants and widespread vaccination efforts, SARS-CoV-2 infection continues to cause hospitalization and mortality in the current post-pandemic era. The present study focused on CD39 and CD73 ectonucleotidases as potential biomarkers, given their relevance to the immuno-thrombotic mechanisms of COVID-19.

Ectonucleotidases are membrane bound enzymes that play a key role in regulating the concentrations of purine nucleotides and nucleosides within the cellular environment, thereby modulating nucleotide- and nucleoside-mediated cellular responses. Nucleoside triphosphate diphosphohydrolase-1 (NTPDase1 or CD39) hydrolyzes ATP and ADP into AMP while ecto-5’-nucleotidase (NT5e or CD73) hydrolyzes AMP into adenosine. Through their sequential hydrolysis, nucleotides - acting as damage-associated molecular patterns - are converted into adenosine, a molecule with anti-inflammatory, anti-thrombotic and vasorelaxant properties^7^. In this way, ectonucleotidases regulate the immune response and suppress thrombosis and inflammation, processes central to the pathophysiology of SARS-CoV-2 infection^8–10^. Notably, ectonucleotidases are predominantly expressed by the vascular endothelium and leukocytes, both of which play key roles in COVID-19-associated coagulopathy and immune dysfunction^11,12^.

Previous studies have demonstrated that the quantification of the soluble ectonucleotidase activity holds prognostic value in various cardio-vascular pathologies, including atherosclerosis or coronary artery disease^13,14^, and is also associated with the pro-inflammatory tumor environment^15,16^. However, research on purinergic signaling in SARS-CoV-2 infection remains limited and inconsistent, likely due to methodological discrepancies. Most studies have detected ectonucleotidase expression on the surface of lymphocyte subpopulations by flow cytometry^17–19^ with circulating forms measured less frequently via ELISA^20,21^. In this study, we aimed to quantify the soluble activity of CD39 and CD73 in the serum of COVID-19 patients and investigate their potential association with disease severity and clinical phenotypes such as hypoxemia and inflammation.

## Methods

### Study population

**Fig. 1:**
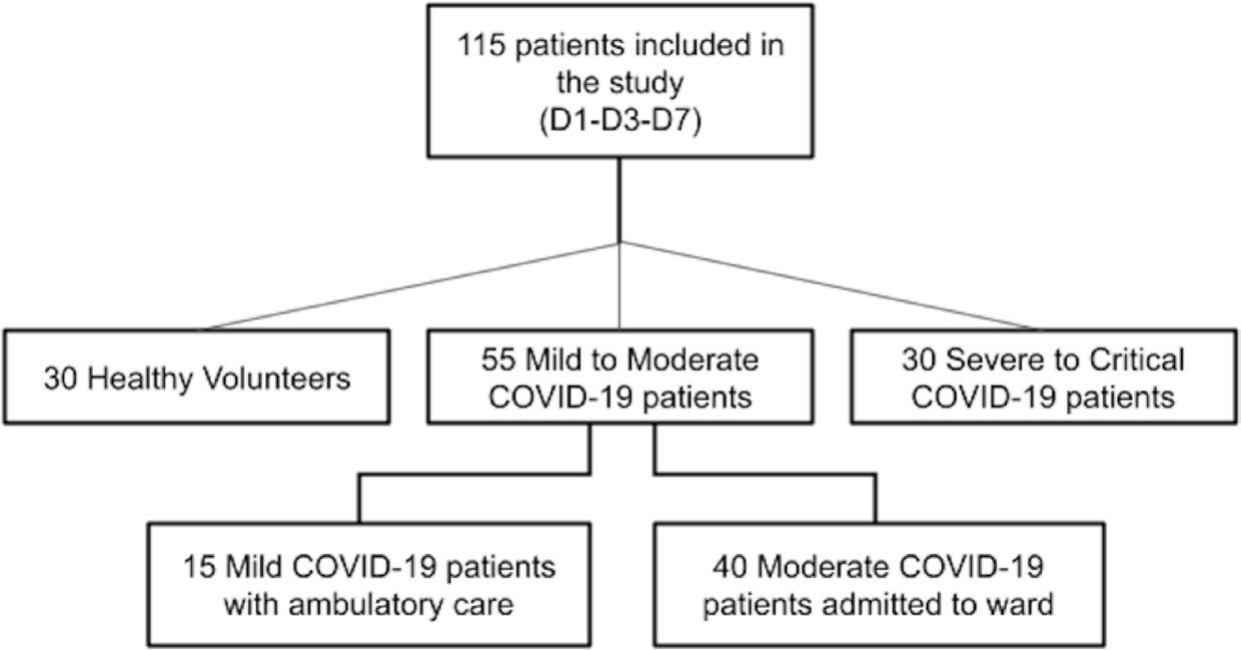
Flowchart of the study population. Among the *n* = 40 moderate patients, only four were admitted to the ICU for a mean duration of 2 ± 1 days, without requiring mechanical ventilation. *Abbreviations*: D = Day, COVID-19 = Coronavirus disease 2019.

The cohort study was conducted at Strasbourg University Hospital, between August 1 st, 2020, and August 1 st, 2021. COVID-19 patients were enrolled, with infection confirmed by a positive reverse transcriptase-polymerase chain reaction (RT-PCR) test on nasal swab. Patients were stratified based on disease severity, according to World Health Organization (WHO) guidelines^22^. The mild COVID-19 group included patients with any symptoms. The moderate COVID-19 group included patients with evidence of lower respiratory disease during clinical assessment or imaging, and those who had an oxygen saturation (SpO_2_) ≥ 94% on room air. The severe to critical COVID-19 group included patients with SpO_2_ <94%, a ratio of arterial partial pressure of oxygen to fraction of inspired oxygen (PaO_2_/FiO_2_) < 300 mmHg, lung infiltrates ≥ 50%, respiratory failure or multiple organ failure. For analysis, patients were further categorized into two severity subgroups: mild to moderate (MM) and severe to critical (SC). Healthy volunteers (HV), matched for sex and age, were recruited from the ED staff, all confirmed by a negative RT-PCR result at the time of sampling (Fig. 1).

### Ethics

The study was approved by the Strasbourg University Hospital Ethics Committee and registered on clinicalTrials.gov (NCT02391792). All procedures involving human participants were performed in accordance with the ethical standards of the institutional research committee and with the 1964 Declaration of Helsinki and its later amendments or comparable ethical standards. Written informed consent was obtained from all participants (both infected and healthy volunteers) or from their next of kin prior to enrollment. Enrolled patients received treatment according to international guidelines applicable at the time of inclusion.

### Data collection

Medical records were reviewed for demographic, clinical and biochemical data, which were standardized in a report file. Data were collected at all three stages (days one, three and seven) of the infection. Recorded variables included main epidemiological factors (age and sex), along with essential comorbidities such as obesity, (body mass index ≥ 30 kg/m^2^), diabetes and a history of cardiovascular disease. All aspects of ED management were documented, including clinical parameters and early organ support strategies, such as endotracheal intubation. Routine laboratory measurements (arterial blood gas, creatinine, CRP) and radiological findings, mainly focusing on the extension of lung parenchyma lesions, were recorded. Ventilation strategy, corticosteroid and anticoagulant treatments, secondary complications, length of stay in the ICU and in-hospital mortality were also documented. Arterial oxygen partial pressure to fractional inspired oxygen (PaO_2_/FiO_2_) was measured within the first twenty-four hours after ICU admission. ARDS was classified according to the Berlin definition^23^.

Two distinct clinical profiles were categorized at admission to the ED (day-1):

1/Hypoxemia-like phenotype defined by a respiratory rate (RR) ≥ 20/min and an oxygen requirement ≥6 L/min to maintain saturation ≥ 95%.

2/Inflammation-like phenotype defined by a temperature ≥ 38 °C and an elevated CRP ≥ 100 mg/L.

#### Blood collection

Blood samples were obtained upon patient admission to the ED (day-1), then on day-3 and day-7. Sampling was performed by venipuncture. After an initial purge (3 ml), blood was collected in 5 ml tubes dry tubes (reference: 367614, volume 5 mL) and centrifuged at 2500 g for 15 min at 20 °C within the hour to obtain serum. The serum was aliquoted and immediately stored at −80 °C until activity measurements. Healthy volunteers were sampled only once.

#### Measurement of CD39/CD73 activities

Serum CD39 and CD73 enzymatic activities were measured as previously described^24^. The enzymatic reaction was performed in Hank’s Balanced Salt Solution (COR21-022-CV) containing 10 mM HEPES, 2 mM CaCl_2_, and 1 mM MgCl_2_, adjusted to pH 7.5 and supplemented with Ap5a (sc-214457, Santa Cruz Biotechnology, Dallas, TX, USA), an inhibitor of adenylate kinase (80 µg/ml only for CD39 activity) and levamisole, an inhibitor of alkaline phosphatase (1 mg/ml). The enzymatic reaction was performed in a final volume of 200 µL at 37 °C: 1 h for CD73 activity and 2 h for CD39 activity, in the presence of patient serum (20 µl for CD73 and 40 µl for CD39 activity measurements). The reaction was initiated by adding etheno-ADP or etheno-AMP (100 µM final) fluorescent derivative as substrates.

Hydrolysis was stopped by adding 10% perchloric acid. After 20 min on ice, samples were centrifuged (15000 g, 10 min, 4 °C) and a fraction of the supernatant was diluted (1/100) in water for HPLC determination. Etheno-ADP, AMP and adenosine were separated on a C18 reversed-phase column (Kromasil 100-5 C18 728041 46, Macherey-Nagel, Düren, Germany), using KH2PO4 50 mM pH 4 as a mobile phase with a methanol gradient. Detection was performed with a fluorescent detector (F-1000 fluorescence spectrophotometer, Hitachi Ltd., Tokyo, Japan) using 280 nm and 410 nm as excitation/emission wavelength respectively. ADPase activity was calculated by measuring fluorescent etheno-ADP hydrolysis. Activities were expressed as pmol of substrate hydrolysed/min/µL of serum after subtracting the reference condition performed without serum.

#### Statistical analysis

Descriptive statistical analysis was performed for the categorical variables by reporting the frequencies and proportions of each value, and for quantitative variables, by reporting the median with the first and third quartiles. Continuous covariates were compared using Student’s t- tests or ANOVA, with Wilcoxon or Kruskal-Wallis tests applied in cases of non-normality. Categorical covariates were compared using Chi-squared or Fisher’s exact tests. Correlation coefficients were calculated for continuous covariates. The association between each independent factor and serum CD73 activity at day-1 ($\:\ge\:$85 pmol/min/µL) were assessed individually using a univariable logistic regression model. Variables included in the multivariable logistic regression model were selected based on clinical relevance and univariate results using a purposeful-selection strategy (entry *p* < 0.20, retention *p* < 0.10). Results were expressed as odds ratios (OR) with their 95% confidence intervals (95% CI). Analyses were performed using GraphPad Prism version 8 (GraphPad Software, San Diego, CA, USA) and R software version 4.3.1 (R Foundation for Statistical Computing, Vienna, Austria).

## Results

### Study population

During the study period, spanning the second and the third waves of the pandemic, 85 patients with SARS-CoV-2 infection and 30 healthy volunteers (HV) were included. Among the infected patients, the vaccination rate was 10.6% at the time of inclusion. In total, 30 patients were classified as having severe to critical disease (SC) and were admitted to the ICU, while 55 patients were classified as having mild to moderate disease (MM). Within the MM subset, 15 patients with mild COVID-19 were managed in ambulatory care and 40 patients with moderate COVID-19 were admitted to the ward (Fig. 1).

### Patients characteristics

Patient characteristics are summarized in Table 1. The median age of MM, SC and HV patients did not differ (52, 61 and 54 years, respectively). The duration of symptom onset was approximately one week in both MM and SC subsets (7 vs.8 days, *p* = 0.179). As previously reported, a larger proportion of SC had a history of hypertension (46.7% vs. 30.9%, *p* = 0.043), obesity (53.3% vs. 32.7%, *p* = 0.023) or diabetes (46.7% vs. 18.2%, *p* < 0.001) compared to MM patients. Severe to critical patients also presented more signs of respiratory distress upon admission with a higher respiratory rate (27/min vs. 22/min, *p* < 0.001), higher oxygen requirement to maintain 95% saturation (92% vs. 95%, *p* < 0.001), higher FiO_2_ (0.50 vs. 0.21, *p* < 0.001) and subsequently lower PaO_2_/FiO_2_ at 24 h (138 vs. 312, *p* < 0.001). Lung parenchymal lesions on CT scan (≥ 25%) were more extended in SC patients compared to MM patients (83.3% vs. 43.4%, *p* < 0.001). White blood cells at day-1 were higher in the SC subset (total leukocytes count: 7920 vs. 6100/µL, *p* = 0.02, neutrophils count: 5525 vs. 4440/µL, *p* = 0.018). Similarly, CRP was higher in SC patients (SC: 119 vs. MM: 65 mg/L, *p* = 0.002). Most patients received anticoagulants (SC: 100% vs. MM: 72.7%, *p* = 0.002) and corticosteroids (SC: 96.7% vs. MM: 63.6%, *p* < 0.001) during their hospital stay. SC patients also had longer hospital stays and duration of oxygen therapy (20 days vs. 5 days, *p* < 0.001). In-hospital mortality was low in our study and therefore it did not differ between the two subgroups, (SC: 13.3% vs. MM: 9.1%, *p* = 0.714). Overall, these findings align with most of the epidemiological reports during the second and the third waves of the pandemic^25^.

**Table 1:** Characteristics and management of the study population.

Demographics	Healthy volunteers *n* = 30	Mild to moderate COVID-19 *n* = 55	Severe to critical COVID-19 *n* = 30	*p* value
Age (years)	54 [47–59]	52 [41–69]	61 [52–69]	0.171
Male	16 (53.3)	33 (60)	22 (73.3)	0.263
**Comorbidities**				
BMI $\:\ge\:$30 (kg/m^2^)	6 (20.0)	18 (32.7)	16 (53.3)	0.023*
Hypertension	5 (16.7)	17 (30.9)	14 (46.7)	0.043*
Diabetes mellitus	1 (3.3)	10 (18.2)	14 (46.7)	< 0.001*
Cardiovascular diseases	5 (16.7)	14 (25.5)	5 (16.7)	0.511
**ED admission (Day-1)**				
Time since 1 st symptom (days)	/	7 [4.5–8.5]	8 [7–10]	0.179
Respiratory rate (/min)	/	22 [20–24]	27 [24–33]	< 0.001*
First O_2_ saturation (%)	/	95 [93–97]	92 [90–95]	0.008*
O_2_ requirement (L/min)	/	0 [0–2]	15 [3–15]	< 0.001*
Fi0_2_	/	0.21 [0.21–0.29]	0.50 [0.40–0.60]	< 0.001*
PaO_2_/FiO_2_ (at 24 h)	/	312 [273–373]	138 [111–181]	< 0.001*
Sp0_2_/FiO_2_	/	443 [333–452]	177 [149–215]	< 0.001*
Temperature (°C)	/	37.9 [37.2–38.3]	38 [37.5–38.5]	0.143
CT extension ($\:\ge\:$25%)	/	23 (43.4)	25 (83.3)	< 0.001*
**Laboratory findings at Day-1**				
Creatinine (µmol/L)	/	64 [56–80.5.5]	62.3 [53.9–83.9]	0.366
Total leukocytes (/µL)	/	6100 [4738–7642]	7920 [4755–11200]	0.020*
Neutrophils (/µL)	/	4440 [3353–6178]	5525 [3530–8478]	0.018*
Lymphocytes (/µL)	/	1035 [723–1190]	625 [458–900]	0.785
NLR	/	5.3 [3.1–7.4]	9 [6.2–17.8]	< 0.001*
Platelets	/	195 [157–253]	203 [160–283]	0.450
CRP (mg/L)	/	65 [34–124]	119 [81–185]	0.002*
D-Dimer (ng/ml)	/	790 [533–1350]	685 [505–1310]	0.598
AST (UI/L)	/	38 [23–62]	35 [24–56]	0.886
ALT (UI/L)	/	34 [24–54]	42 [30–64]	0.143
**Hospital stay**				
Number of days under O_2_	/	5 [0–7]	20 [15–29]	< 0.001*
Corticosteroids	/	35 (63.6)	29 (96.7)	< 0.001*
Anticoagulant therapy	/	40 (72.7)	30 (100)	0.002*
Oro-tracheal intubation	/	4 (7.3)	29 (96.7)	< 0.001*
ARDS	/	5 (9.1)	25 (83.3)	< 0.001*
LOS (days)	/	5 [0–7]	20 [15–29]	< 0.001*
ICU Stay	/	12 (21.8)	30 (100)	< 0.001*
In-hospital mortality	/	5 (9.1)	4 (13.3)	0.714

Data are all expressed as median [Q1-Q3] or n/N (%) where n is the total number of patients with available data. Comparisons of proportions were carried out with chi-squared or Fisher tests and comparisons of continuous covariates were carried out t- or Wilcoxon tests. * *p* < 0.05.

*Abbreviations*: COVID-19 = Coronavirus disease 2019, BMI= Body mass index, ED= Emergency Department, O_2_= Oxygen, FiO_2_= Fraction of inspired oxygen, h= hour, PaO_2_= partial pressure of arterial oxygen, SpO_2_= Oxygen saturation, CT= Computer tomography, D = Day, NLR= Neutrophil to lymphocyte ratio, CRP = C reactive protein, AST= aspartate transaminase, ALT= alanine aminotransferase, ARDS= acute respiratory distress syndrome, LOS= length of stay, ICU= intensive care unit.

### Serum CD73 activity is increased in COVID-19 patients

**Fig. 2:**
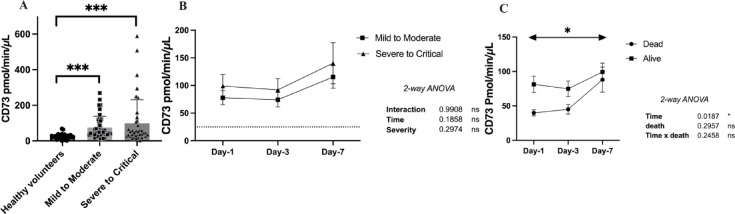
Serum CD73 activity according to COVID-19 severity. CD73 activity according to disease severity (A-B) and according to in-hospital mortality (C). Data are all expressed as mean ± SEM where n is the total number of patients with available data. Statistical analysis: Kruskal-Wallis test (A) and 2 Way-ANOVA (B-C), * *p* < 0.05, ** *p* < 0.01, *** *p* < 0.001. *Abbreviations*: pmol= picomol, min=minute, µL= microliter, ns = non significant.

Serum CD73 activity was significantly increased in COVID-19 patients compared to HV, both for the MM and SC subsets (HV: 25 ± 3 vs. MM: 76 ± 9, SC: 99 ± 21 pmol/min/µL, *p* < 0.0001) (Fig. 2 A). However, the difference in CD73 activity between MM and SC patients did not reach statistical significance at day-1 (78 ± 13 vs. 99 ± 21 pmol/min/µL, *p* = 0.44), day-3 or day-7 (Fig. 2B). Serum CD73 activity was further analyzed according to in-hospital mortality. A total of 9 patients (10.5%) died during their hospital stay, and their CD73 activity at day-1 was among the lowest, although statistical significance was not achieved (non-survivors: 39 ± 4 pmol/min/µL vs. survivors: 81 ± 12 pmol/min/µL). However, non-survivors showed a significant rise in CD73 activity (non-survivors: 45 ± 7 and 88 ± 18 pmol/min/µL, vs. survivors: 74 ± 11 and 99 ± 13 pmol/min/µL, day-3 and day-7, respectively) (Fig. 2 C). Furthermore, CD73 activity at day-1 was significantly correlated with oxygen therapy requirements to achieve saturation ≥ 95% (*r* = 0.285, *p* = 0.008), but was not correlated with other clinical parameters and biomarkers. Noteworthy, liver dysfunction related enzymes were correlated with CD73 activity (AST: *r* = 0.526, *p* < 0.001 and ALT: *r* = 0.570, *p* < 0.001), consistent with prior literature^14^. (Supplemental data Table 1).

### Serum CD73 activity is higher in COVID-19 patients with a hypoxemia-like phenotype

**Fig. 3:**
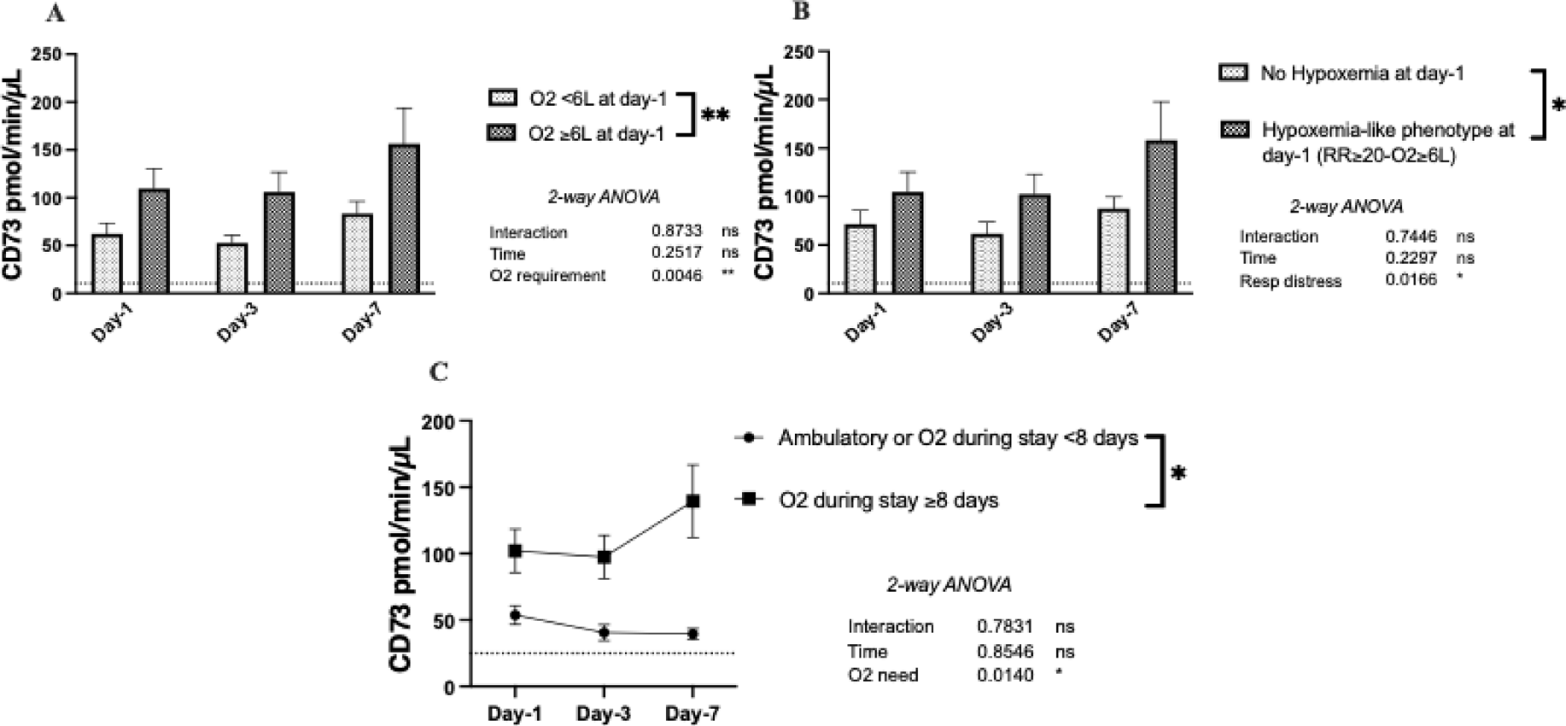
Serum CD73 activity according to hypoxemia. CD73 activity according to O_2_ requirement in the ED (A), according to hypoxemia-like phenotype at day-1 (B) and according to O_2_ requirement during the hospital stay (C) Data are all expressed as mean ± SEM where n is the total number of patients with available data. Statistical analysis: 2 Way-ANOVA (A-B-C), * *p* < 0.05, ** *p* < 0.01, *** *p* < 0.001. *Abbreviations*: pmol= picomol, min=minute, µL= microliter, L=liter, RR=respiratory rate, O2 = oxygen, ns = not significant, ED= Emergency Department.

Upon admission to the ED (day-1), patients requiring oxygen therapy ≥6 L/min exhibited increased serum CD73 activity, persistent over time (109 ± 21 vs. 62 ± 11 pmol/min/µL, *p* = 0.005) (Fig. 3 A). Similarly, patients presenting a hypoxemia-like phenotype (RR$\:\ge\:$20/min and O_2_ requirement $\:\ge\:$6 L/min) had significantly higher CD73 activity (105 ± 20 vs. 71 ± 14 pmol/min/µL, *p*=0.02) (Fig. 3B). Moreover, ambulatory patients and those requiring a short oxygen therapy (< 8 days) had significantly lower CD73 activity (54 ± 7 vs. 102 ± 16 pmol/min/µL, *p*=0.01) compared to patients requiring prolonged oxygen therapy (≥ 8 days) during their hospital stay (Fig. 3 C).

### Serum CD73 activity is lower in COVID-19 patients with an inflammation-like phenotype

**Fig. 4:**
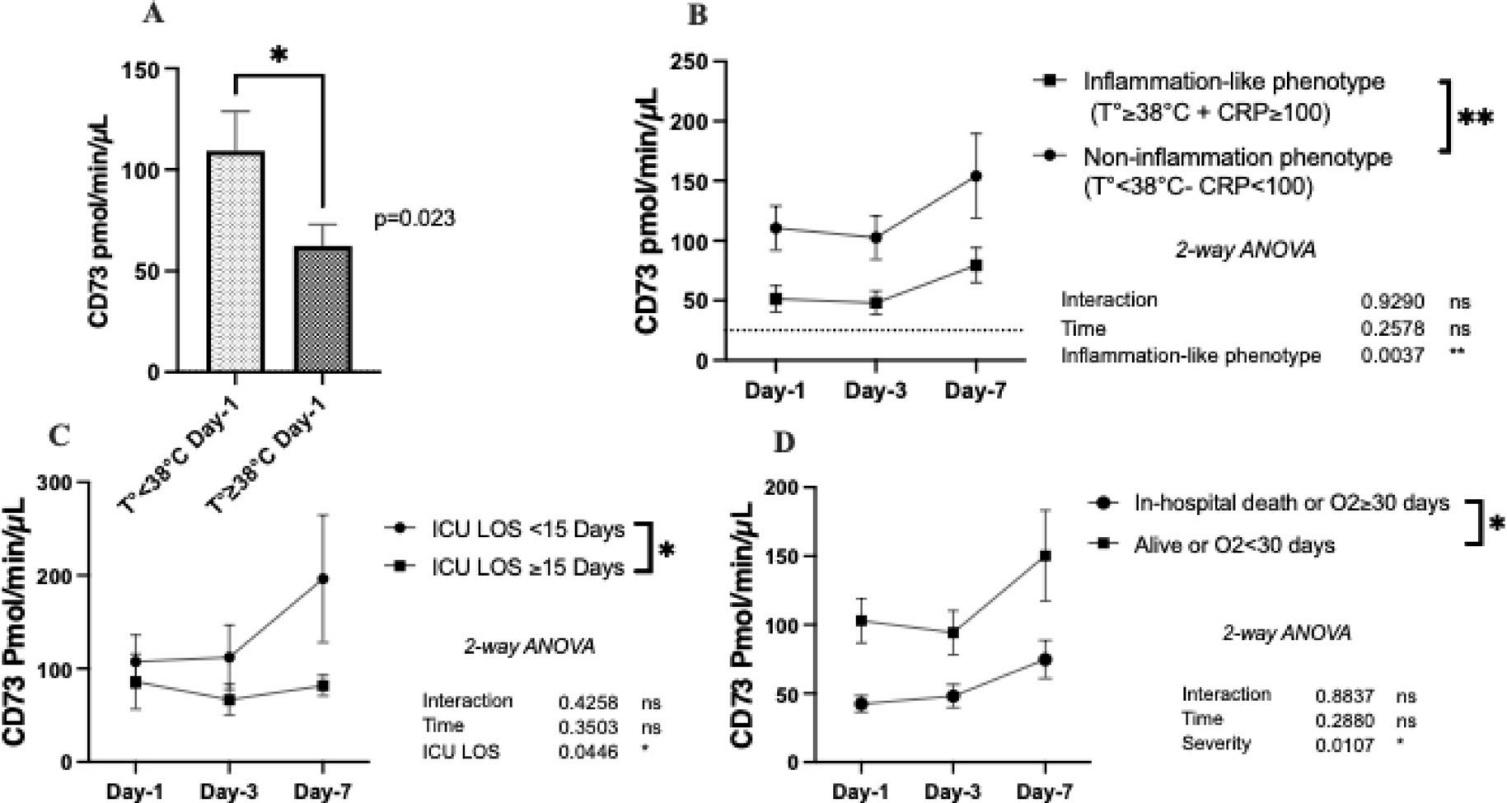
Serum CD73 activity according to inflammation and outcome. CD73 according to temperature at day-1 (**A**), according to inflammation-like phenotype (**B**), according to ICU LOS (**C**) and according to in hospital-mortality (**D**). Data are all expressed as mean ± SEM where n is the total number of patients with available data. Statistical analysis: Mann et Whitney test (A) and 2 Way-ANOVA (B-C-D), * *p* < 0.05, ** *p* < 0.01, *** *p* < 0.001. *Abbreviations*: pmol= picomol, min=minute, µL= microliter, L=liter, CRP = c reactive protein, O2 = oxygen, LOS= length of stay, ICU= intensive care unit, ns = not significant.

At day-1, febrile patients (T°≥38 °C) displayed lower serum CD73 activity compared to apyretic patients (62 ± 11 vs. 109 ± 20 pmol/min/µL, *p* = 0.023). Similarly, patients with an inflammation-like phenotype (T°≥38 °C and CRP ≥ 100 mg/L), had significantly lower CD73 activity at day-1 (51 ± 11 vs. 110 ± 19 pmol/min/µL), day-3 (47 ± 10 vs. 102 ± 18 pmol/min/µL) and day-7 (80 ± 15 vs. 154 ± 35 pmol/min/µL) compared to patients presenting temperatures and CRP levels below these thresholds (*p* = 0.004) (Fig. 4B).

### Serum CD73 activity as a prognosis marker for disease severity and mortality

In SC patients, CD73 activity was associated with the length of the stay (LOS) in the ICU. At day-7, CD73 activity was elevated in SC patients with shorter LOS, compared to SC patients (LOS < 15 days: 196 ± 68 vs. LOS ≥ 15 days 81 ± 11 pmol/min/µL, *p* = 0.044). Conversely, patients who died during their hospital stay or those with prolonged hospital stay (LOS ≥ 30 days), exhibited significantly lower and stable CD73 activity consistently from day-1 to day-7 (day-1: 42 ± 6 vs. 103 ± 16 pmol/min/µL, *p* = 0.01) (Fig. 4 C-D).

### High CD73 activity is associated with severe clinical phenotype at day-1

 Independent factors associated with high CD73 activity at day-1 (≥ 85 pmol/min/µL) were investigated using univariable and multivariable analysis. Univariable analysis identified three independent conditions significantly associated with high CD73 activity at day-1: a major oxygen therapy requirement above 6 L/min (OR = 2.60, CI95%: 0.98 to 7.21, *p* = 0.04), indicating over two folds increase in the likelihood of high CD73 activity; a temperature over 38 °C (OR = 0.27, CI95%: 0.09 to 0.74, *p* = 0.01); and an inflammation-like phenotype (OR = 0.19, CI95%: 0.03 to 0.72, *p* = 0.03), both associated with a significant decrease in the likelihood of high CD73 activity. Multivariable analysis confirmed the inverse association between the inflammation-like phenotype and high CD73 activity, which remained significant (OR = 0.12, CI95%: 0.02 to 0.52, *p* = 0.01). Although not reaching statistical significance, data suggested that patients with a hypoxemia-like phenotype were almost nine times more likely to display elevated CD73 activity at day-1 (OR = 8.76, 95% CI: 1.10 to 123.66, *p* = 0.06). No other clinical parameters were significantly associated with CD73 (Table 2 and supplementary material, Table S2).

**Table 2:** Univariate and Multivariate analysis of the factors associated with High CD73 at Day-1 ($\:\ge\:$85 pmol/min/µL).

	Univariate				Multivariate			
	Odds Ratio	CI (2.5)	CI (97.5)	*p*-value	Odds Ratio	CI (2.5)	CI (97.5)	*p*-value
Age	0.98	0.95	1.01	0.21	0.97	0.93	1.01	0.19
Female	0.96	0.34	2.61	0.95				
RR at admission	1.03	0.96	1.11	0.37				
O_2_ for SpO_2_ $\:\ge\:$95%	1.06	0.98	1.16	0.14				
O_2_$\:\ge\:$6 L/min	2.60	0.98	7.31	0.04*				
Hypoxemia-like phenotype	2.30	0.88	6.31	0.10	8.76	1.10	123.66	0.06
CT Extension ($\:\ge\:$25%)	3.63	0.62	69.14	0.23				
Temp $\:\ge\:$38 °C	0.27	0.09	0.74	0.01*				
CRP $\:\ge\:$100 mg/L	0.93	0.35	2.44	0.89				
Inflammation-like phenotype	0.19	0.03	0.72	0.03*	0.12	0.02	0.52	0.01*
Oro-tracheal intubation	1.30	0.49	3.45	0.59				
Extended duration O_2_ ($\:\ge\:$7D)	1.94	0.72	5.67	0.20	1.47	0.26	7.86	0.65
ICU stay	1.62	0.60	4.32	0.34	1.61	0.19	17.99	0.67
Extended Hospital stay ($\:\ge\:$10D)	1.77	0.68	4.83	0.25	2.42	0.33	18.26	0.38
ICU LOS	0.99	0.95	1.02	0.57				

Hypoxemia-like phenotype: RR$\:\ge\:$20/min et O_2_$\:\ge\:6$L/min.

$$\:\mathrm{I}\mathrm{n}\mathrm{f}\mathrm{l}\mathrm{a}\mathrm{m}\mathrm{m}\mathrm{a}\mathrm{t}\mathrm{i}\mathrm{o}\mathrm{n}-\mathrm{l}\mathrm{i}\mathrm{k}\mathrm{e}\:\mathrm{p}\mathrm{h}\mathrm{e}\mathrm{n}\mathrm{o}\mathrm{t}\mathrm{y}\mathrm{p}\mathrm{e}:\:\mathrm{T}^\circ\:\ge\:38^\circ\:\mathrm{C}\hspace{0.17em}+\hspace{0.17em}\mathrm{C}\mathrm{R}\mathrm{P}\ge\:100\mathrm{m}\mathrm{g}/\mathrm{L}$$.

*Abbreviations*: CI= Confidence interval, RR= Respiratory rate, SpO_2_= Oxygen saturation, CT= Computer tomography, CRP = C reactive protein, D= Days, ICU= Intensive Care Unit, LOS= length of stay, T°= Temperature, L= liter, pmol= picomol, min=minute, µL= microliter.

### Serum CD39 activity is not associated with COVID-19 nor with disease severity

 In the study population, CD39 activity could only be measured in 71.8% of patients at day-1, while values were below the detection limit in 28.2% of patients. Possible factors contributing to this CD39 distribution were explored by comparing clinical data and biological markers. A history of obesity and hypertension was more frequent in patients with undetectable CD39 activity (obesity: 58.3% vs. 32.8%, *p* = 0.03; hypertension: 54.2% vs. 29.5%, *p* = 0.034), consistent with previous reports (Supplementary Data, Table S1)^13,14^. In addition, in patients with a history of hypertension (no hypertension: 11.8 ± 1.8 vs. hypertension: 19.7 ± 2.2 pmol/min/µL, *p* = 0.04) or obesity (non-obesity: 12% vs. obesity: 41.2%, *p* = 0.02), circulating CD39 (when measurable) was significantly lower than in those without such history. No other clinical or biochemical parameter was significantly associated with CD39 activity (Supplemental material, Table S3).

At day-1, CD39 activity was similar in all three patient subgroups (HV: 12.4 ± 2.1, MM: 18.5 ± 2.5 and SC: 15.4 ± 2.2 pmol/min/µL, *p* = 0.264, Supplementary Data, Figure S1A). In addition, CD39 was also non-discriminatory at day-3 or day-7, with values remaining comparable at any time point (*p* = 0.581, Supplementary Data, Figure S1B).

## Discussion

SARS-CoV-2 infection exemplifies a systemic disease in which severity is primarily driven by respiratory failure leading to ARDS, sometimes accompanied by cytokine storm or thrombotic events^26^. In this context, ectonucleotidases CD39 and CD73, through nucleotide hydrolysis and adenosine generation, are involved in regulating platelet aggregation, cytokine secretion and neutrophil extracellular trap (NET) release - key contributors to severe COVID-19 manifestations^27^.

In this study, we evaluated the serum enzymatic activities of CD39 and CD73 as potential biomarkers of COVID-19 severity, measured at three time points (days 1, 3, and 7). Our results show that CD73 activity increases in hypoxemic patients and decreases in those with an inflammation-like phenotype, reflecting distinct profiles that may be relevant for patient management in the ED. Indeed, the identification of reliable biomarkers remains a challenge in SARS-CoV-2 infection, with three main objectives: early diagnosis, accurate risk stratification for triage, and monitoring of treatment response^4^.

### CD73 activity, but not CD39 activity, as a predictor of COVID-19 severity

COVID-19 is clinically heterogeneous, with some patients developing a hyperinflammatory state whereas others exhibit endothelial dysfunction or thrombotic complications^28^. Furthermore, distinct immunotypes observed in critically ill patients may underlie the heterogeneous clinical trajectories and outcomes^29^. In the present work, analyzing three serial samples per patient helped account for infection timing and disease progression, thereby reducing interindividual variability.

Our findings notably revealed an inverse association between CD73 activity and the inflammation-like phenotype, which closely aligns with the immunological profile of patients likely to benefit from immunomodulatory treatments. Hence, CD73 activity might serve as a useful marker to guide therapeutic decisions in this subgroup, although potential variations under immunomodulatory treatment could not be assessed due to the small number of treated patients.

Yet, compared with previous reports, our data, derived from a larger cohort of 85 COVID-19 patients, reveal a positive association between CD73 activity and disease severity, particularly hypoxemia, a crucial parameter for initial triage in the ED. By contrast, CD39 activity remained unchanged.

Several studies have evaluated CD73 activity during SARS-CoV-2 infection, yielding conflicting results^17,18^. *Da Silva et al.* measured circulating nucleotide concentrations, CD39 and CD73 expression in peripheral blood mononuclear cells (PBMC) by flow cytometry in 62 COVID-19 patients. They reported increased ATP concentrations, decreased ATP hydrolysis and increased AMP hydrolysis along with elevated CD39 and CD73 protein expression in PBMCs, suggesting that the serum CD73 activity we quantified may originate from PBMCs. In contrast, another study of 28 patients reported decreased plasma CD73 activity and adenosine deaminase during SARS-CoV-2 infection, correlating with disease severity and the occurrence of thrombosis^20^.

Notably, in the present study, CD39 activity was undetectable in the serum of more than a quarter of the cohort, with no association between CD39 activity and disease severity. This suggests that circulating CD39 activity does not fully mirror its membrane expression on vascular cells, primarily endothelial and leukocyte populations. Interestingly, CD39 particularly expressed by Treg lymphocytes and M2 macrophages, contributes to their immunosuppressive function by accelerating nucleotide hydrolysis and adenosine generation. Despite reports of aberrant overexpression of CD39 on exhausted T-cells obtained at autopsy from COVID-19 patients, correlating to vasculopathy^30^, elevated CD39 plasma concentrations have also been associated with hypoxemia in COVID-19 patients^21^. Altogether, these reports suggest a complex relationship between the different CD39 reservoirs, tissular or vascular, depending on patient’s evolving immune status throughout the course of the infection. In our study, CD39 activity was not deemed a relevant biomarker for assessing patients’ immune status or infection severity.

### Drivers and Mechanisms of CD73 Elevation in SARS-CoV-2 Infection

We demonstrated a significant and positive association between CD73 activity and oxygen requirement on day 1, which persisted throughout hospitalization. This finding is consistent with hypoxia-induced CD73 upregulation mediated by HIF-1α, which increases adenosine production and supports tissue protection under low-oxygen conditions^31,32^. Interestingly, both mild ambulatory patients and non-survivors displayed low serum CD73 activity. This duality likely reflects the regulation of purinergic signaling throughout the disease course: low CD73 levels in mild cases may represent an early phase before hypoxia-driven upregulation, whereas reduced activity in fatal cases may signal exhaustion of this compensatory pathway under sustained inflammation. Because differences in mortality were not statistically significant and specific causes of death were unavailable, this association warrants cautious interpretation. A plausible integrated explanation is that CD73 upregulation under hypoxic conditions enhances adenosine production, favoring adaptation to hypoxia and limiting tissue injury, as previously reported in high-altitude adaptation and inflammatory injury^33,34^.

In the present work, patients presenting an inflammation-like phenotype exhibited a specific pattern with significantly low serum CD73 activity on day-1 which remained persistently low throughout their hospital stay. A plausible explanation is that CD73 participates in the control of inflammation by generating adenosine. Consequently, an enhanced expression of CD73 would increase adenosine levels^8^, as previously described in *Leishmania amazonensis* infection^35^. The initial rise in adenosine would contribute to an immunosuppressive and anti-thrombotic microenvironment, modulating host responses while potentially favoring viral proliferation and entry into target cells^36^. Indeed, single cell multiomics profiling in nasopharyngeal swabs and blood from individuals challenged with pre-Alpha SARS-CoV2, revealed that, at day 3, the interferon response in blood preceded the immune infiltration observed on day 7 in the nasopharynx, with further delay in the case of sustained infection. This supports the hypothesis that CD73 storage pools may vary depending on the timing of infection and the sequence of events triggered by the virus^37^. Yet, our findings support the view that CD73 activity reflects the balance between early adaptation and late inflammatory exhaustion during the first week in the ED.

### Relevance of Circulating Ectonucleotidase Activity Compared With PBMC Antigenic Expression

Most studies have focused on the cell surface-tethered forms of these enzymes, an active form of CD73 carried by small vesicles being nevertheless reported in the plasma of untreated patients with head and neck squamous carcinoma^38^. Our method is highly relevant for detecting membrane-bound active ectonucleotidase directly in patients’ serum, unlike previous studies that analyzed various subsets of circulating cells using indirect immunological probing, a method strongly dependent on antibody quality. Furthermore, our direct quantification approach offers a robust, reproducible and less complex preanalytical sampling technique, which is particularly valuable in the context of a pandemic.

Interestingly, our observations differ from those of Madera-Sandoval et al.^39^, who reported higher proportions of CD73-positive B-like cells in non-surviving patients. However, their analysis relied on the percentage of CD73-positive PBMCs rather than on quantitative expression or enzymatic activity, and CD73 showed no prognostic value in their study. These methodological differences likely explain the divergence with our results, as the presence of CD73 on immune cells does not necessarily reflect its circulating catalytic function. The respective contribution of immune-cell reservoirs to serum CD73 activity across inflammatory states remains to be clarified. Of note, modulation of the adenosinergic pathway has been explored as a therapeutic strategy in COVID-19^40,41^, and regadenoson, an A2A receptor agonist, has shown anti-inflammatory effects in experimental models and in a small number of patients^42^.

### Serum CD73: origin and activity

CD73 is a GPI (glycosylphosphatidylinositol)-anchored protein susceptible to proteolytic cleavage by specific phospholipases, allowing its release as a truly soluble ectoenzyme in peripheral blood^43^. The full GPI-anchored CD73 can also circulate when carried by plasma membrane vesicles (MVs) shed from activated cells. In our assay, both the truncated and membrane-derived forms present in blood may account for the measured serum activity. Yet, the respective contribution of these two circulating forms in COVID-19, as well as the identity of their cellular sources, which are likely multiple, remains to be clarified. Overall, the elevated CD73 activity observed in our cohort likely reflects immune dysfunction - a hallmark of COVID-19 - and may represent part of the host response to mitigate inflammation and vascular damage.

### Implications and pathophysiological insights

Although viral variants and the vaccination landscape have evolved since the early pandemic, identifying robust biomarkers to stratify disease severity remains a major challenge. Our study, conducted during the first epidemic wave in a SARS-CoV-2–naïve population, offers insights into the early immunometabolic responses associated with COVID-19 severity. Altered CD73 activity, shaped by hypoxia and inflammation - both defining features of severe disease - indicates that purinergic signaling contributes to the host response and drives vascular and immune dysregulation. Given the increasing recognition of post-acute sequelae of SARS-CoV-2 infection (long COVID), stable and mechanistically anchored biomarkers such as CD73 may also aid in predicting long-term outcomes^44^.

Overall, a multi-marker approach integrating clinical, biochemical, imaging, and immunological parameters appears essential to enhance early risk stratification^45^. Such a strategy could not only improve the sensitivity and specificity of severity prediction but also assist in identifying patients eligible for targeted therapies. Larger and longitudinal cohorts remain essential to consolidate the prognostic relevance of CD73 and delineate its cellular origins and mechanistic pathways.

## Limitations

This study evaluated the potential value of serum ectonucleotidase activities as biomarkers of disease severity in SARS-CoV-2 infection. However, several limitations should be acknowledged. First, this was a single-center study with a relatively modest sample size, which may limit the statistical power and generalizability of the findings. Nonetheless, in comparison with other studies, our cohort ranks among the largest to date, and we were able to collect longitudinal enzymatic data throughout hospitalization allowing consistent temporal assessment. Second, the timing of patient inclusion may have introduced potential biases related to evolving treatment protocols and changes in standards of care. However, most patients received corticosteroids and intensified anticoagulation, in accordance with WHO recommendations in place during the study period. Finally, because only a small proportion of patients received immunomodulatory therapy (*n* = 8 Tocilizumab, *n* = 1 Anakinra), their impact on ectonucleotidase activities could not be evaluated.

## Conclusion

Serum CD73 activity is increased in COVID-19, particularly in hypoxemic patients, whereas persistently low activity characterizes the inflammation-like phenotype and unfavorable outcomes. This pattern likely reflects early hypoxia-driven activation followed by exhaustion under sustained inflammation, supporting CD73 as a dynamic biomarker of COVID-19 severity.

## Supplementary Information

Below is the link to the electronic supplementary material.

Supplementary Material 1

## Data Availability

All data generated or analysed as part of the study are included.

## Abbreviations

COVID-19: Coronavirus disease-19
ED: Emergency Department
ICU: Intensive care unit
ARDS: acute respiratory distress syndrome
CRP: C-reactive protein
MM: mild to moderate
SC: severe to critical
HV: healthy volunteers
LOS: length of stay
