# Supplementary material for: Serum CD73 activity as a biomarker of hypoxemia in COVID-19 patients
Source: Sci Rep. 2026 Feb 26;16:11080. doi: 10.1038/s41598-026-41023-2 (PMC13043897; doi:10.1038/s41598-026-41023-2)
Supplement: Supplementary file 1 — Supplementary Material 1 [file 41598_2026_41023_MOESM1_ESM.docx]

**Supplementary material**

**Table S1: Comparing detectable and undetectable serum CD39 activity in patients with COVID-19: clinical and laboratory characteristics**

| **Demographics** | **CD39=0 at Day-1**  **n=24** | **CD39>0 at Day-1**  **n=61** | **p value** |
| --- | --- | --- | --- |
| Age (years) | 63 [45-69] | 56 [42-68] | 0.682 |
| Male | 16 (66.7) | 39 (63.9) | 0.812 |
| **Comorbidities n (%)** |  |  |  |
| BMI $\geq$30 (kg/m^2^) | 14 (58.3) | 20 (32.8) | **0.030*** |
| Hypertension | 13 (54.2) | 18 (29.5) | **0.034*** |
| Diabetes mellitus | 10 (41.7) | 14 (23) | 0.084 |
| Cardiovascular diseases | 5 (20.8) | 14 (23) | 0.833 |
| **ED management (Day-1)** |  |  |  |
| Time since 1^st^ symptom (days) | 7 [4-8] | 8 [5-10] | 0.165 |
| Respiratory rate (/min) | 24 [22-27] | 22 [20-26] | 0.187 |
| First O_2_ saturation (%) | 92.5 [91-95] | 94 [91-96] | 0.129 |
| O_2_ requirement (L/min) | 6 [2-11] | 3 [0-9] | 0.058 |
| Fi0_2_ admission | 0.37 [0.21-0.54] | 0.25 [0.21-0.50] | 0.269 |
| PaO_2_/FiO_2_ (at 24h) | 219 [140-296] | 300 [143-367] | 0.062 |
| Temperature (°C) | 38 [37.5-38.6] | 38 [37.2-38.3] | 0.315 |
| CT extension ($\geq$25%) | 17 (70.8) | 31 (52.5) | 0.126 |
| **Laboratory findings Day-1** |  |  |  |
| Creatinine (μmol/L) | 65 [58-81] | 63 [54-81] | 0.729 |
| Total leukocytes (/μL) | 6195 [5175-8918] | 6645[4577-8480] | 0.593 |
| Neutrophils (/μL) | 5035 [3908-6950] | 4845 [3225-7288] | 0.334 |
| Lymphocytes (/μL) | 785 [530-1083] | 890 [568-1183] | 0.303 |
| NLR | 7.1 [5.4-9.4] | 6.3 [3.1-9.6] | 0.158 |
| Platelets | 210 [182-268] | 192 [155-258] | 0.135 |
| CRP (mg/L) | 92 [57-170] | 85 [40-146] | 0.507 |
| D-dimer (ng/mL) | 1085 [580-1780] | 710 [520-1085] | 0.114 |
| AST (UI/L) | 24 [22-46] | 40 [28-59] | 0.081 |
| ALT (UI/L) | 37 [27-60] | 37 [26-61] | 0.965 |
| **Hospital stay** |  |  |  |
| Number of days under O_2_ | 8 [5-19] | 7 [0-18] | 0.205 |
| Corticosteroids | 22 (91.7) | 42 (68.9) | **0.028*** |
| Anticoagulant therapy | 23 (95.8) | 47 (77.1) | 0.068 |
| Oro-tracheal intubation | 11 (45.8) | 22 (36.1) | 0.406 |
| ARDS | 10 (41.7) | 20 (32.8) | 0.441 |
| LOS (days) | 13 [7-32] | 8 [3-23] | 0.196 |
| ICU Stay | 14 (58.3) | 28 (45.9) | 0.305 |
| In-hospital mortality | 3 (12.5) | 6 (9.8) | 0.988 |
|  |  |  |  |

Data are all expressed as median [Q1-Q3] or n/N (%) where n is the total number of patients with available data. * p <0.05.

Comparisons of proportions were performed using chi-squared or Fisher tests, and comparisons of continuous covariates were carried out t- or Wilcoxon tests.

Abbreviations: BMI= Body mass index, ED= Emergency Department, O_2_= Oxygen, FiO_2_= Fraction of inspired oxygen, h= hour, PaO_2_= partial pressure of arterial oxygen, SpO_2_= Oxygen saturation, CT= Computer tomography, D= Day, NLR= Neutrophil to lymphocyte ratio, CRP= C reactive protein, AST= aspartate transaminase, ALT= alanine aminotransferase, ARDS= acute respiratory distress syndrome, LOS= length of stay, ICU= intensive care unit.

**Figure S1: Serum CD39 activity according to COVID-19 severity**

CD39 activity according to disease severity (A-B).

Data are expressed as mean ±SEM where n represents the number of patients with available data. Statistical analysis: Kruskal-Wallis test (A) and 2 Way-ANOVA (B), * p <0.05, ** p<0.01, *** p<0.001.

Abbreviations: pmol= picomol, min=minute, µL= microliter, ns=not significant

**Table S2: Correlation between circulating CD73 and clinical parameters at ED admission, laboratory findings and outcome**

| **Variables** | **Correlation coefficient** |  | **p value** |
| --- | --- | --- | --- |
| Age | -0.125 [-0.301-0.060] |  | 0.184 |
| Time since 1^st^ symptom | 0.070 [-0.145-0.279] |  | 0.524 |
| **ED admission (Day-1)** |  |  |  |
| RR | 0.204 [-0.010-0.399] |  | 0.062 |
| O_2_ need for saturation $\geq$95% | 0.285 [0.076-0.469] |  | **0.008*** |
| Fi0_2_ | 0.182 [-0.032-0.381] |  | 0.095 |
| PaO_2_/FiO_2_ | -0.025 [-0.246-0.199] |  | 0.828 |
| Temperature | -0.174 [-0.373-0.041] |  | 0.112 |
| **Laboratory findings (Day-1)** |  |  |  |
| Creatinine | -0.042 [-0.253-0.173] |  | 0.704 |
| Total leukocytes | 0.016 [-0.199-0.229] |  | 0.887 |
| Neutrophils | -0.029 [-0.242-0.186] |  | 0.792 |
| Lymphocytes | -0.015 [-0.229-0.200] |  | 0.891 |
| NLR | -0.029 [-0.242-0.186] |  | 0.792 |
| Platelets | -0.116 [-0.323-0.101] |  | 0.293 |
| CRP | -0.049 [-0.260-0.166] |  | 0.654 |
| D-dimer | -0.071 [-0.286-0.151] |  | 0.196 |
| AST | 0.526 [0.335-0.675] |  | **<0.001*** |
| ALT | 0.570 [0.391-0.707] |  | **<0.001*** |
| **Outcome** |  |  |  |
| Number of days under O_2_ | -0.020 [-0.194-0.232] |  | 0.857 |
| Number of days in the ICU | 0.053 [-0.162-0.263] |  | 0.631 |
| Duration of MV | 0.002 [-0.213-0.216] |  | 0.986 |
| Total LOS | 0.173 [-0.042-0.373] |  | 0.113 |

Abbreviations: RR= Respiratory rate, ED= Emergency Department, O_2_= Oxygen, FiO_2_= Fraction of inspired oxygen, h= hour, PaO_2_= partial pressure of arterial oxygen, SpO_2_= Oxygen saturation, D= Day, NLR= Neutrophil to lymphocyte ratio, CRP= C reactive protein, AST= aspartate transaminase, ALT= alanine aminotransferase, MV= Mechanical ventilation, LOS= length of stay, ICU= intensive care unit

**Table S3: Correlation between circulating CD39 and clinical parameters at ED admission, laboratory findings and outcome**

| **Variables** | **Correlation coefficient** | **p value** | | |
| --- | --- | --- | --- | --- |
| Age | 0.007 [-0.209-0.222] | | 0.950 |  |
| Time since 1^st^ symptom | -0.279 [-0.496-0.029] | | **0.029*** |  |
| **ED admission (Day-1)** |  | |  |  |
| RR | -0.065 [-0.312-0.190] | | 0.616 |  |
| O_2_ need for saturation $\geq$95% | -0.086 [-0.331-0.170] | | 0.510 |  |
| Fi0_2_ | -0.092 [-0.336-0.164] | | 0.481 |  |
| PaO_2_/FiO_2_ | 0.004 [-0.262-0.269] | | 0.978 |  |
| Temperature | -0.016 [-0.267-0.237] | | 0.902 |  |
| **Laboratory findings (Day-1)** |  | |  |  |
| Creatinine | -0.044 [-0.293-0.210] | | 0.735 |  |
| Total leukocytes | -0.115 [-0.358-0.143] | | 0.382 |  |
| Neutrophils | -0.150 [-0.389-0.108] | | 0.253 |  |
| Lymphocytes | 0.134 [-0.125-0.375] | | 0.309 |  |
| NLR | -0.174 [-0.410-0.083] | | 0.183 |  |
| Platelets | -0.057 [-0.307-0.200] | | 0.664 |  |
| CRP | -0.156 [-0.393-0.100] | | 0.229 |  |
| D-dimer | -0.052 [-0.307-0.209] | | 0.696 |  |
| AST | 0.149 [-0.133-0.407] | | 0.298 |  |
| ALT | 0.216 [-0.063-0.464] | | 0.128 |  |
| **Outcome** |  | |  |  |
| Number of days under O_2_ | -0.071 [-0.317-0.184] | | 0.588 |  |
| Number of days in the ICU | -0.002 [-0.254-0.250] | | 0.987 |  |
| Duration of MV | 0.039 [-0.217-0.290] | | 0.765 |  |
| Total LOS | -0.055 [-0.303-0.199] | | 0.673 |  |

Abbreviations: RR= Respiratory rate, ED= Emergency Department, O_2_= Oxygen, FiO_2_= Fraction of inspired oxygen, h= hour, PaO_2_= partial pressure of arterial oxygen, SpO_2_= Oxygen saturation, D= Day, NLR= Neutrophil to lymphocyte ratio, CRP= C reactive protein, AST= aspartate transaminase, ALT= alanine aminotransferase, MV= Mechanical ventilation, LOS= length of stay, ICU= intensive care unit
